# Clearing Amyloid-Beta by Astrocytes: The Role of Rho GTPases Signaling Pathways as Potential Therapeutic Targets

**DOI:** 10.3390/brainsci14121239

**Published:** 2024-12-10

**Authors:** Gyeongah Park, Zhen Jin, Hui Lu, Jianyang Du

**Affiliations:** 1 Department of Anatomy and Neurobiology, University of Tennessee Health Science Center, Memphis, TN 38163, USA; gpark4@uthsc.edu; 2 Department of Pharmaceutical Sciences, University of Tennessee Health Science Center, Memphis, TN 38163, USA; zjin6@uthsc.edu; 3 Department of Pharmacology and Physiology, School of Medicine, The George Washington University, Washington, DC 20037, USA; huilu@email.gwu.edu

**Keywords:** Alzheimer’s disease, amyloid-β (Aβ), Aβ clearance, Rho GTPases, astrocytes, endothelin-converting enzyme, neprilysin

## Abstract

Astrocytes, vital support cells in the central nervous system (CNS), are crucial for maintaining neuronal health. In neurodegenerative diseases such as Alzheimer’s disease (AD), astrocytes play a key role in clearing toxic amyloid-β (Aβ) peptides. Aβ, a potent neuroinflammatory trigger, stimulates astrocytes to release excessive glutamate and inflammatory factors, exacerbating neuronal dysfunction and death. Recent studies underscore the role of Rho GTPases—particularly RhoA, Rac1, and Cdc42—in regulating Aβ clearance and neuroinflammation. These key regulators of cytoskeletal dynamics and intracellular signaling pathways function independently through distinct mechanisms but may converge to modulate inflammatory responses. Their influence on astrocyte structure and function extends to regulating endothelin-converting enzyme (ECE) activity, which modulates vasoactive peptides such as endothelin-1 (ET-1). Through these processes, Rho GTPases impact vascular permeability and neuroinflammation, contributing to AD pathogenesis by affecting both Aβ clearance and cerebrovascular interactions. Understanding the interplay between Rho GTPases and the cerebrovascular system provides fresh insights into AD pathogenesis. Targeting Rho GTPase signaling pathways in astrocytes could offer a promising therapeutic approach to mitigate neuroinflammation, enhance Aβ clearance, and slow disease progression, ultimately improving cognitive outcomes in AD patients.

## 1. Introduction

### 1.1. Astrocytes and Aβ Clearance

Astrocytes, the most abundant glial cell type in the central nervous system (CNS), play a pivotal role in maintaining neuronal health and function [1]. They are in close contact with cerebral blood vessels and neurons through their foot process structures, forming a key network across the blood–brain barrier, and play an indispensable role in maintaining the homeostasis of the CNS. These cells not only provide nutritional support for neurons, but also regulate ion balance, secrete neurotrophic factors, and play an important role in removing metabolic waste [2,3,4,5]. Their diverse functions include providing structural support, regulating ion homeostasis, and participating in neurotransmitter clearance. Increasing evidence suggests that astrocytes actively contribute to the pathogenesis of multiple neurological disorders. In particular, astrocytes have an important role in waste removal processes because their foot process structures form glial limitans around cerebral blood vessels, helping to remove the accumulation of harmful substances such as amyloid β protein (Aβ) [6,7]. Aβ is one of the key pathological features of Alzheimer’s disease (AD) and is usually accumulated significantly in neurodegenerative lesions [8]. In 2012, Jeffrey et al. showed that astrocytes effectively limit the spread of Aβ in the brain parenchyma by regulating the fluidity of cerebrospinal fluid and brain interstitial fluid, thereby playing a vital role in regulating the metabolic balance of Aβ in the brain [9]. In recent years, increasing evidence has highlighted the significance of astrocytes in the clearance of Aβ peptides, a hallmark of AD [10,11,12,13].

#### 1.1.1. Astrocyte Reactivity and Functions

Morphological changes: reactive astrocytes often exhibit hypertrophy and hyperplasia, accompanied by increased expression of glial fibrillary acidic protein (GFAP), a hallmark marker of astrocyte activation [14]. These morphological changes can lead to the formation of glial scars, which may impede neuronal regeneration and plasticity [15,16].

Altered gene expression: reactive astrocytes upregulate the expression of a variety of genes, including inflammatory cytokines, chemokines, and growth factors [17,18,19,20]. Some of these factors, such as interleukin-1β (IL-1β) and tumor necrosis factor-α (TNF-α), can exacerbate neuroinflammation and contribute to neurodegeneration [21,22,23]. However, other factors, such as brain-derived neurotrophic factor (BDNF) and glial cell line-derived neurotrophic factor (GDNF), may have neuroprotective effects [24,25].

Functional modifications: reactive astrocytes can exhibit altered ion channel and transporter expression, leading to changes in their ability to regulate extracellular ion concentrations and neurotransmitter levels [26,27,28,29]. They may also release neurotoxic substances, such as glutamate and reactive oxygen species (ROS), which can damage neurons [30,31].

#### 1.1.2. Astrocyte-Mediated Aβ Clearance Mechanisms

Phagocytosis: astrocytes can directly engulf and degrade Aβ through phagocytosis. This process involves the recognition of Aβ by specific receptors on the astrocyte surface, followed by internalization and lysosomal degradation [32,33,34].

Endocytosis: astrocytes can internalize Aβ through endocytosis, a process that involves the formation of vesicles that transport Aβ into the cell [35,36,37]. Once inside the cell, Aβ can be degraded by lysosomal enzymes or transported to the perivascular space for clearance [38,39].

Perivascular clearance: astrocytes play a critical role in perivascular clearance, a process by which Aβ is transported from the brain parenchyma to the cerebrospinal fluid (CSF) via the perivascular space. Astrocytes extend their endfeet, to wrap around blood vessels, forming a tight junction that regulates the exchange of substances between the blood and the brain [9]. Aβ can be transported from the brain parenchyma to the perivascular space through these endfeet and then cleared into the CSF [40].

Secretion of Aβ-degrading enzymes: astrocytes can secrete a variety of enzymes, including neprilysin, insulin-degrading enzyme (IDE), and matrix metalloproteinases (MMPs), that can degrade Aβ. These enzymes can cleave Aβ into smaller, less toxic fragments, thereby reducing its neurotoxicity [12,41,42].

#### 1.1.3. Impaired Astrocyte Function and Aβ Accumulation

In AD, astrocyte function becomes impaired, leading to reduced Aβ clearance and increased Aβ accumulation [43]. This impairment may be due to a variety of factors, including oxidative stress, inflammation, and genetic mutations [18,44,45]. As a result, Aβ accumulates in the brain parenchyma, forming plaques that disrupt neuronal communication and contribute to neurodegeneration.

#### 1.1.4. Therapeutic Implications

Understanding the role of astrocytes in Aβ clearance has important therapeutic implications for AD. By targeting astrocyte function, it may be possible to enhance Aβ clearance and slow disease progression. Potential therapeutic strategies include the following: 

Pharmacological interventions: developing drugs that can enhance astrocyte function, such as by increasing the expression of Aβ-degrading enzymes or reducing inflammation [46].

Cell therapy: transplanting healthy astrocytes into the brain to replace damaged or dysfunctional astrocytes [47]. Using gene therapy to deliver genes that can enhance astrocyte function or reduce Aβ production [48]. 

In conclusion, astrocytes play a crucial role in Aβ clearance, and impaired astrocyte function contributes to Aβ accumulation and AD pathogenesis. By understanding the mechanisms underlying astrocyte-mediated Aβ clearance, we may be able to develop novel therapeutic strategies for AD.

### 1.2. Rho GTPases in Astrocyte

#### 1.2.1. Rho GTPases in Cellular Functions

Rho GTPases, a family of small GTPases, are molecular switches that cycle between an inactive GDP-bound state and an active GTP-bound state. This dynamic regulation allows them to control a wide range of cellular processes, including cell proliferation, differentiation, migration, and adhesion [49]. They are particularly crucial in regulating the actin cytoskeleton, a complex network of proteins that provides structural support to cells and enables various cellular functions, such as cell motility and division [50].

One of the most critical roles of Rho GTPases lies in the regulation of the actin cytoskeleton, a dynamic network of actin filaments that provides structural support to cells and enables various cellular functions, such as cell motility, division, and morphogenesis. Rho GTPases influence actin dynamics through several mechanisms: 

Actin polymerization: Rho GTPases, particularly Ras homolog family member A (RhoA) and Rac1, can directly interact with actin-binding proteins, such as the Arp2/3 complex, to promote actin polymerization [51,52]. This leads to the formation of actin filaments, which are essential for cell shape and motility [53]. 

Stress fiber formation: RhoA is a key regulator of stress fiber formation, which produces contractile bundles of actin filaments [54]. The activation of RhoA leads to the activation of Rho-associated kinase (ROCK), which phosphorylates the myosin light chain, promoting myosin–actin interactions resulting in stress fiber formation [55,56]. 

Lamellipodia and filopodia formation: Ras-related C3 botulinum toxin substrate 1 (Rac1) and cell division control protein 42 homolog (Cdc42), respectively, are key regulators of lamellipodia and filopodia formation [57,58,59]. These membrane protrusions are essential for cell migration and invasion. Activation of Rac1 and Cdc42 leads to the activation of various actin-binding proteins, such as WASP and WAVE, which promote actin polymerization and the formation of these protrusions [60,61]. 

Endocytosis and exocytosis: Rho GTPases also play a role in regulating endocytosis and exocytosis, processes that involve the formation and fusion of membrane-bound vesicles. For example, Rac1 and Cdc42 are involved in clathrin-mediated endocytosis, while RhoA is involved in exocytosis [62,63,64,65]. 

#### 1.2.2. Rho GTPases in Diseases

Dysregulation of Rho GTPases has been implicated in a variety of human diseases, including cancer [66], cardiovascular disease, and neurodegenerative disorders. For example, aberrant activation of RhoA has been linked to tumor invasion and metastasis, while aberrant activation of Rac1 has been linked to autoimmune diseases [67,68]. In neurodegenerative diseases, such as AD and Parkinson’s disease, dysregulation of Rho GTPases has been implicated in neuronal cell death and synaptic dysfunction [69,70]. Given their critical role in various cellular processes and their involvement in numerous diseases, Rho GTPases have emerged as attractive therapeutic targets. Several strategies are being explored to target Rho GTPases, including the following: 

Small-molecule inhibitors: these compounds can directly inhibit Rho GTPases or their downstream effectors [71,72]. 

RNA interference: RNA interference can be used to knock down the expression of Rho GTPases [73]. For example, lentiviral vector-mediated shRNA targeting RhoA was applied to cultured Schwann cells to suppress RhoA expression [74]. 

Gene therapy: gene therapy can be used to overexpress or downregulate Rho GTPases. The designed vector, such as bacterial enzyme C3-ADP ribosyl transferase (C3), blocks RhoA from becoming active and helps axons grow and regenerate, which promotes outgrowth [75]. By targeting Rho GTPases, it may be possible to develop novel therapies for a wide range of diseases. However, further research is needed to fully understand the complex roles of Rho GTPases in cellular processes and to develop safe and effective therapies.

#### 1.2.3. Rho GTPases in the CNS

In the central nervous system (CNS), Rho GTPases play a pivotal role in neuronal development, synaptic plasticity, and neurodegenerative diseases [76,77,78]. They regulate several key processes, including the following:

Axon guidance and growth cone dynamics: in the nervous system, Rho GTPases play a pivotal role in neuronal development and regeneration by regulating the cytoskeleton, with particular importance in dendrite and axon growth [79,80,81,82,83,84]. During axon guidance, Rho, Rac, and Cdc42 GTPases serve as key modulators of neuroplasticity. RhoA activation increases myosin II activity, resulting in axon retraction and growth cone collapse. Conversely, Rac1 and Cdc42 promote axon extensions by driving the formation of lamellipodia and filopodia. Through these mechanisms, Rho GTPase signaling regulates actin filament dynamics in growth cones, enabling them to respond effectively to guidance cues [85]. 

Rho GTPases also mediate growth cone responses to neurotrophic factors. For instance, the activation of Trk and p75 receptors can either prompt actin filament aggregation or reduce RhoA activity, thereby shaping the pseudopodia structures of growth cones [86,87]. Highly conserved in eukaryotes, Rho GTPases primarily control neuronal migration by orchestrating the assembly and actin rearrangement and microtubule cytoskeletons. Rho GTPases regulate cell polarity, adhesion, and directional migration during the formation of cortical neuron layers and the patterning of brain circuits, facilitating the development of cortical structure and establishing functional neural connections [88]. These roles make Rho GTPases indispensable as cytoskeletal regulators in nervous system development.

Dendritic spine morphology and synaptic plasticity: Rho GTPases influence the formation, maturation, and elimination of dendritic spines, which are the sites of synaptic contact between neurons. They also regulate synaptic plasticity, the ability of synapses to strengthen or weaken in response to activity. The Rho family of small GTPases are essential regulators of synaptic plasticity, influencing synaptic development through actin cytoskeleton remodeling. Key members, including Rac1, Cdc42, and RhoA, along with their effectors, play crucial roles in spine formation, spine morphology, receptor trafficking, and the processes underlying synaptic plasticity, learning, and memory. The activation of Rac1 and Cdc42 leads to an increase in immature spines. Some of these spines mature via a Rac1-dependent pathway, while others are pruned through a RhoA-dependent mechanism. This supports selective growth and balanced synaptic regulation [89]. 

In the active state, Rho GTPases engage with downstream effectors to orchestrate spine morphology and synapse development [90]. Studies have identified various GEFs and GAPs within the Rho family as essential for spine morphogenesis. Rho GTPases are regulated by GEFs (guanine nucleotide exchange factors), which facilitate the exchange of GDP for GTP to activate the GTPases, and by GAPs (GTPase-activating proteins), which accelerate GTP hydrolysis to inactivate them [90]. Within the spine, GEFs are pivotal in regulating the actin cytoskeleton by modulating Rho GTPase activity. The activation of Rac and Cdc42 supports the formation and growth of synapses and spines, whereas RhoA restrains excessive synaptic development, maintaining a dynamic balance in excitatory synapses [13]

Neuroinflammation: Rho GTPases are involved in the inflammatory response in the CNS, regulating the activation of microglia and astrocytes. Inflammation in the central nervous system (CNS) activates the small GTPase RhoA and its downstream effector ROCK. Activation of this pathway not only directly leads to neuronal damage and cell death, but also promotes the shrinkage and loss of neural processes and synapses [91]. In the CNS inflammatory response, the participation of astrocytes and microglia exacerbates neuronal damage, and the RhoA/ROCK signaling pathway plays a key role in regulating the functions of these glial cells and immune cells. Studies have shown that the activation of the RhoA/ROCK pathway induces neurodegeneration in the CNS [92,93].

Under neuroinflammatory conditions, heparan sulfate proteoglycans (CSPGs) released by astrocytes interact with myelin-associated inhibitor Nogo receptors, activating the RhoA/ROCK pathway and leading to axonal growth cone collapse [94]. Studies by Fujita et al. showed that the activation of RhoA/ROCK induces cytoskeletal reorganization, inhibiting neurite growth and leading to growth cone collapse [95]. In contrast, the inhibition of the RhoA/ROCK pathway promotes neurite regeneration and recovery. This was further verified by Zhang et al.’s study: by inhibiting ROCK activity with Fasudil, neuronal recovery and a significant reduction in the proliferation of reactive astrocytes after cerebral ischemia/reperfusion injury were observed [96,97]. 

In summary, the RhoA/ROCK pathway plays an important role in neuronal injury and neurodegeneration by regulating glial cell activation and neurotoxic phenotype transition in CNS inflammation. Inhibiting this pathway not only helps reduce neuronal damage caused by neuroinflammation but also promotes neuronal regeneration and CNS repair.

#### 1.2.4. Rho GTPases in Regulating Astrocyte Morphology

Rho GTPases are essential regulators of astrocyte morphology, function, and reactivity. Holtje et al. found that Rho plays an inhibitory role in astrocyte neurite formation during astrocyte stellation [98]. By selectively inhibiting Rho’s downstream effector ROCK with Y27632, they observed accelerated wound healing, enhanced polarized neurite formation, and increased astrocyte migration toward the lesion site, suggesting that Rho negatively regulates astrocyte neurite growth and migration responses after injury [90]. Etienne-Manneville et al. further showed that Rho influences the microtubule cytoskeleton during astrocyte migration, while Rac is essential for neurite development and maintenance in migrating astrocytes [99]. Additionally, Cdc42 is critical for forming neurites that contribute to the elongated morphology of astrocytes [99]. This implies that Rac facilitates cell elongation by directly affecting microtubule dynamics or modulating microtubule-dependent processes. Supporting this, Daub et al. showed that Rac may regulate microtubule dynamics by phosphorylating p65PAK, which inhibits the microtubule-destabilizing protein [100].

These findings collectively suggest that Rho, Rac, and Cdc42 have distinct roles in astrocyte morphology and migration, with Rho acting as a negative regulator, while Rac and Cdc42 contribute to neurite elongation and stability by modulating the microtubule cytoskeleton. 

#### 1.2.5. Rho GTPases in Regulating Astrocyte Function and Reactivity

Rho GTPases also regulate a variety of astrocyte functions, including the following:

Gliotransmitter release: astrocytes release gliotransmitters, such as glutamate and ATP, which can modulate neuronal activity. Rho GTPases have been identified as key regulators of exocytosis and may play a role in modulating the exocytosis of these gliotransmitters [101,102]. 

Water and ion homeostasis: astrocytes play a crucial role in maintaining water and ion homeostasis in the brain [103,104]. Rho GTPases regulate the expression and activity of ion channels and transporters, which are essential for this function [105,106]. 

Neuroinflammation: as mentioned earlier, Rho GTPases are involved in the inflammatory response in the CNS, including the activation of astrocytes. Inhibition of the RhoA/ROCK pathway can significantly reduce reactive gliosis, reduce the over-activation of astrocytes, and induce their expression of pro-survival genes [107,108,109,110]. Profilin 1 (PFN1), as one of the downstream effectors of RhoA/ROCK, may play a neuroprotective role by affecting the polarization of microglia. Ermei et al. indicated that knocking down PFN1 can promote the neuroprotective polarization of microglia, which may be achieved through the inhibition of RhoA/ROCK [111,112]. In addition, NF-κB, a downstream effector of ROCK, plays an important role in the neurotoxic phenotype conversion of microglia. Zhang et al. found that inhibiting the RhoA/ROCK/NF-κB pathway can prevent microglia from polarizing to neurotoxic subtypes, promoting their transformation into neuroprotective phenotypes, and help them recover from brain damage under inflammatory conditions [113]. This activation can lead to the release of inflammatory cytokines and chemokines, which may contribute to neurodegeneration.

In response to injury or disease, astrocytes undergo a process known as reactive astrogliosis. This process involves changes in astrocyte morphology, gene expression, and function. Rho GTPases play a critical role in regulating reactive astrogliosis [114]. For example, the activation of RhoA can promote the formation of glial scars, which can limit tissue damage but can also hinder neuronal regeneration [115]. In contrast, loss of Rac1 can promote neurogenesis and synaptogenesis [116]. Rho GTPases are essential regulators of astrocyte function and reactivity. By understanding the role of Rho GTPases in astrocytes, we can gain insights into the mechanisms underlying neurodegenerative diseases and develop novel therapeutic strategies. Targeting Rho GTPases may provide a promising approach to modulate astrocyte function and promote neuroprotection. 

### 1.3. Rho GTPases in Aβ Clearance 

Given that Rho GTPases are dysregulated in AD, several studies have investigated the relationship between Rho GTPases, amyloid precursor protein (APP) synthesis, and Aβ production across various cell lines. For instance, in primary hippocampal neurons from mice, the inhibition of Rac1 negatively regulates APP gene synthesis [117] and reduces Aβ42 production by altering γ-secretase substrate selectivity, leading to increased processing of Notch1 instead of APP [118].

The dysregulation of Rho GTPase signaling has been linked to impaired Aβ clearance and the progression of AD. For instance, age-related changes in Rho GTPase activity can contribute to decreased astrocytic and microglial function, leading to diminished Aβ clearance and increased deposition of neurotoxic aggregates. Moreover, genetic and pharmacological modulation of Rho GTPases has shown promise in preclinical models, suggesting that maybe targeting these signaling pathways could enhance Aβ clearance and provide therapeutic benefits in AD [119,120,121].

Thus, in the following sections, we discuss the mechanisms by which astrocyte-associated Rho GTPases facilitate Aβ clearance from the brain, particularly focusing on the role of Rho GTPases in Aβ clearance enzymes in astrocytes. Additionally, we explore the potential of astrocyte Rho GTPases as therapeutic targets for disease modification in AD.

## 2. Aβ Clearance Enzymes in Astrocytes 

In AD, the accumulation of Aβ is a hallmark pathological feature, with astrocytes playing a crucial role in its clearance through various mechanisms. Astrocytes first help reduce Aβ deposition in the brain by transferring Aβ from the brain parenchyma to the perivascular space, a process dependent on the functional integrity of the neurovascular unit [122]. Additionally, Aβ can be transported across the blood–brain barrier (BBB) and cleared out of the brain, helping to mitigate its neurotoxicity [123,124]. Astrocytes also contribute by clearing Aβ from the brain’s lymphatic system, thus reducing Aβ-associated damage through their clearance functions [9,125]. In particular, astrocytes degrade Aβ by secreting proteases, such as neprilysin (NEP) and ECEs (ECE-1 and ECE-2) as Aβ-degrading enzymes. These enzymes cleave Aβ peptides at specific sites, inactivating or transforming them to reduce their toxicity and accumulation [126,127]. 

### 2.1. NEP 

NEP is a key Aβ-degrading enzyme primarily located in hippocampal neurons in the CA1-3 region [128,129] and in reactive astrocytes, but is found less frequently in microglia [130,131]. Studies have shown that elevated NEP levels in AD patients can reduce Aβ 42 [132]. Furthermore, Kim et al. found that exercise-induced hormones significantly increased NEP release in astrocytes, effectively reducing Aβ levels [11]. NMDA antagonists inhibit NEP’s role in Aβ degradation, reducing the ability of astrocytes to manage exogenous Aβ [133]. Other studies have shown that astrocyte transplantation can promote Aβ clearance, while NEP inhibitors can negatively affect Aβ clearance efficiency [10]. However, NEP plays a key role in the clearance of extracellular Aβ, while ECEs primarily degrade Aβ intracellularly [134]. Rho GTPases are molecular switches that relay extracellular signals into the cell, where they initiate intracellular events. 

### 2.2. ECE-1 

Multiple studies provide evidence that ECEs in astrocytes assist Aβ clearance and may protect against AD. Eckman et al. demonstrated that ECE-1 and ECE-2 can degrade Aβ in vitro and in vivo, with reduced ECE activity leading to increased amyloid plaque formation. This was one of the first studies to suggest ECE’s role in regulating Aβ levels in the brain [135]. Iwata et al. confirmed that decreased ECE activity results in Aβ accumulation in animal models [136]. Subsequent research by Padilla et al. and Palmer et al. emphasized astrocyte ECE’s essential role in clearing Aβ from brain capillaries, highlighting astrocyte ECEs as key to Aβ breakdown, particularly in the perivascular space [137,138]. 

ECE-1 is a membrane-bound protein that plays a critical role in mediating vasoconstriction during inflammatory responses or tissue injury. It achieves this by converting large precursor molecules of endothelin-1 (ET-1) into their biologically active forms. ECE-1 is primarily localized in endosomal compartments and has been shown to exhibit activation in endothelial cells in response to Aβ [138,139]. 

**Figure 1:**
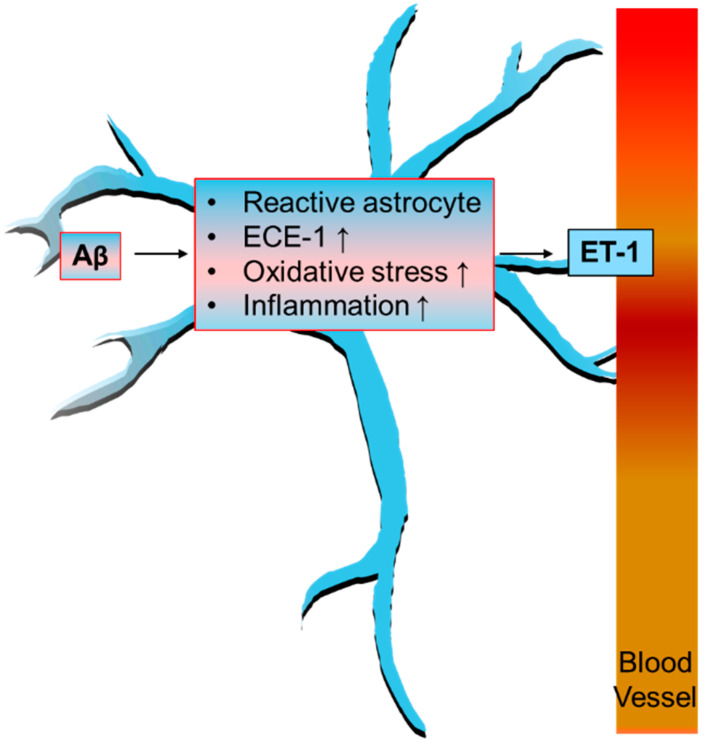
Schematic diagram of astrocyte ECE-1 in Aβ clearance. The upward arrow indicates an increase.

The presence of Aβ oligomers is particularly concerning, as these aggregates can disrupt cerebral blood flow in capillaries. This disruption is mediated through the production of ROS, which subsequently triggers the release of ET-1 [140]. Elevated levels of ET-1 can have detrimental effects on neurovascular function and exacerbate neuroinflammatory processes within the brain. Specifically, overexpression of ET-1 in astrocytes has been linked to heightened neuroinflammatory damage, worsening the overall pathological state [141]. Interestingly, research has indicated that inflammatory responses lead to the activation of reactive astrocytes, particularly in conditions like focal multiple sclerosis lesions, which are identified as primary sources of ET-1. In contrast, astrocytes that remain unaffected do not display significant ET-1 activity [142] (Figure 1). This differential expression underscores the role of reactive astrocytes in the context of neuroinflammation and highlights the complex interplay between neuroinflammatory factors and glial cell responses.

Further investigations into inflammatory mediators, such as interleukin-1 beta (IL-1β), revealed that these cytokines can significantly upregulate ET-1 production in astrocytes. Conversely, compounds like resveratrol showed potential in inhibiting ET-1 production, suggesting promising therapeutic avenues to mitigate neuroinflammation and associated vascular dysfunction [143]. By exploring the pathways involving ECE-1, researchers may uncover new strategies for treating neurodegenerative diseases characterized by inflammation and impaired blood flow.

### 2.3. ECE-2

ECE-2 is predominantly expressed in neurons but is also found in specific populations of astrocytes and microglia [144,145]. This enzyme plays a crucial role in the metabolism of neuropeptides and has been implicated in various neurobiological processes. Studies utilizing ECE-2 knockout mice demonstrated that the absence of this enzyme leads to significantly increased levels of Aβ in the brain. This accumulation of Aβ is closely associated with cognitive impairments, resulting in notable deficits in memory and learning [139,146]. Interestingly, in patients diagnosed with AD, research showed that the levels of ECE-2 mRNA are elevated. This increase may be a compensatory response to the accumulation of Aβ and reduced cerebral blood flow often observed in AD [145]. The relationship between ECE-2 and Aβ levels is complex. While ECE-2 deficiency appears to contribute to elevated Aβ levels, it is essential to consider that other contributing factors may play a more significant role in this process. For instance, the pathological environment created by neuroinflammation and vascular dysfunction in AD may influence the dynamics of Aβ accumulation independently of ECE-2 level [147,148,149,150]. Thus, while the relationship between ECE-2 and Aβ accumulation is noteworthy, it is crucial to recognize that the mechanisms underlying Aβ deposition in AD are multifactorial.

Future research should aim to clarify the precise role of ECE-2 in the context of neurodegenerative diseases and explore potential therapeutic interventions that target this enzyme to mitigate cognitive decline associated with Aβ accumulation. Understanding these intricate pathways could pave the way for novel strategies in the treatment of AD.

Collectively, these studies affirm that ECE-1 and ECE-2, particularly within astrocytes, significantly contribute to Aβ degradation, potentially reducing the risk of amyloid plaque formation in the brain. 

## 3. Rho GTPase Family and Their Signaling Pathways Regulating Aβ Clearance Enzymes 

The Rho GTPase family may effectively regulate the activity of astrocyte ECEs through multiple signaling pathways, affecting the function of the endothelin system. Astrocytes regulate the expression and release of ECEs by releasing proinflammatory factors and oxidative stress molecules, changing the level of endothelin, and playing a key role in the pathological process of the nervous system [151,152,153]. The following are the specific mechanisms and pathological effects of Rho GTPase family members regulating astrocyte ECE and its related signaling pathways. 

### 3.1. RhoA/ROCK

The RhoA/ROCK signaling pathway affects the morphology, adhesion, and activation state of astrocytes by regulating the remodeling of the cytoskeleton. When RhoA activates ROCK, the contraction and reorganization of the cytoskeleton are enhanced, making astrocytes more active under inflammatory or oxidative stress conditions, promoting the release of ECEs [154]. Research by Minamino et al. showed that the enhanced ECE activity can catalyze the production of more endothelin and activate surrounding endothelin receptors, regulate local vascular tension, and aggravate inflammatory responses and ECE-1 may promote the occurrence and development of atherosclerosis through the autocrine and paracrine mechanisms of endothelin-1 and blocking ECE-1 can effectively reduce this promoting effect [155]. 

Studies have shown that inhibiting ROCK can reduce reactive gliosis and increase the expression of astrocyte pro-survival genes [93,98,156,157], and this regulation is essential for the health of the nervous system. Kimura et al. showed that upregulation of the RhoA/ROCK pathway is closely related to a series of pathological processes in ischemic stroke and spinal cord injury [158], including neuronal apoptosis, neuroinflammation, BBB dysfunction, astrogliosis, and axonal growth inhibition. Animal models and clinical trials have demonstrated that that ROCK inhibitors, such as Fasudil and VX-210, can reduce apoptosis, neuroinflammation, oxidative stress, and axonal growth inhibition in ischemic stroke and spinal cord injury [159,160,161].

In addition, inhibiting the RhoA/ROCK pathway may have deleterious effects on neuroinflammation, BBB dysfunction, neuronal apoptosis, astrogliosis, and axonal injury after ischemic stroke. Wen et al. demonstrated that the inhibition of the RhoA/ROCK pathway with Y-27632 can significantly improve cerebral ischemia/reperfusion injury [162]. 

These findings provide a new perspective for understanding the dual role of the RhoA/ROCK signaling pathway in neuropathology and offer potential intervention targets for the future treatment of ischemic injury.

### 3.2. Rac1

The production of brain ET-1, which increases in brain disorders, is involved in the pathophysiological response of the nervous system. Barker et al. have concluded that the brain of AD patients has an increased amount of ET-1 in the temporal cortex of the brain [163]. Rac1 plays an important role in oxidative stress response, mainly by regulating the production of ROS [164]. Rac1 promotes the generation of ROS by activating NADPH oxidase, increasing the oxidative stress level of astrocytes [165,166,167], which may affect the expression and secretion of ECEs [168,169]. At the onset of neuroinflammation, Rac1-mediated ROS generation is vital, as it activates and boosts pro-inflammatory signaling ECE activity, and increases endothelin production [170,171].

Therefore, in neurodegenerative diseases such as AD, the activation of Rac1 may be closely related to the generation of ROS and the upregulation of ECEs in astrocytes, leading to increased endothelin levels, aggravated neuroinflammation, and oxidative stress, thus causing further damage to neurons.

### 3.3. Cdc42

Cell division control protein 42 homolog (Cdc42) plays an important role in astrocyte migration and morphological regulation, especially by controlling the cytoskeleton, and regulating the formation of cell pseudopods and cell extensibility, enhancing the migration ability of astrocytes [172,173]. The activity of Cdc42 can enhance the migration and reactivity of astrocytes [99]. Especially with brain trauma, astrocytes will migrate to the damaged area and release ECEs to regulate local blood vessels and assist in repair. In models of neural injury [174,175] and in patients with stroke, traumatic brain injury, and neurodegenerative diseases, such as AD, brain levels of ET-1 are significantly elevated [176,177,178]. 

Immunohistochemical studies showed that ET-1 in the damaged brain is mainly produced by brain microvascular endothelial cells and reactive astrocytes [179,180]. Studies have shown that factors such as TNF-α, IL-1β, thrombin, and hypoxia can induce brain microvascular endothelial cells and astrocytes to secrete ET-1, and ET-1 itself can also stimulate astrocytes to further produce ET-1 [181,182,183,184]. However, excessive release of ECEs may lead to excessive vasoconstriction, further triggering ischemia and delaying the tissue recovery process. In brain trauma models, the activation of Cdc42 promotes the migration of astrocytes to the injured area and induces vascular responses through the activation of the endothelin system to support the repair of the injured area [185,186,187,188].

**Figure 2:**
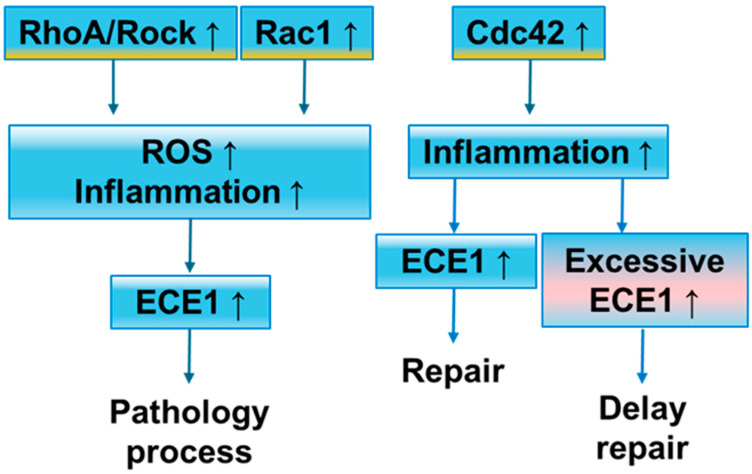
Rho GTPase family’s role with endothelin-converting enzyme 1 in astrocytes. The upward arrow represents an increase.

In summary, Rho GTPase family members RhoA, Rac1, and Cdc42 regulate the activity of ECEs and the endothelin system in astrocytes through multiple signaling pathways, affecting vascular regulation and inflammatory response. These regulatory mechanisms show specific patterns in different pathological states, revealing the potential target value of Rho GTPase signaling in neuropathological processes (Figure 2).

## 4. Conclusions

The role of Rho GTPases in the central nervous system has received increasing attention, especially their regulatory role in astrocyte function and the mechanisms of neurodegenerative diseases. Astrocytes are the most abundant glial cells in the central nervous system and play an important role in maintaining homeostasis, providing metabolic support to neurons, and clearing neurotoxic substances such as Aβ. 

The accumulation of Aβ is a major feature of AD and is directly related to the significant neurodegenerative process. The complex interactions between Rho GTPases, astrocyte ECE activity, and Aβ clearance reveal the high complexity of astrocyte responses in neurodegenerative diseases. Studying the dynamic regulatory mechanisms of these signaling pathways may not only deepen the understanding of astrocyte function, but also provide novel therapeutic strategies to enhance Aβ clearance. 

Studying how Rho GTPases regulate the activity of astrocytes will not only help to reveal these mechanisms, but also may provide potential therapeutic targets for AD and similar diseases. RhoA, Rac1, and Cdc42 in the Rho GTPase family are the focus of research, which significantly affects the morphology and function of astrocytes. These GTPases maintain the structural integrity and plasticity of astrocytes by regulating cytoskeletal dynamics. The RhoA/ROCK signaling pathway has been shown to promote astrocyte activation, enhance the secretion of proinflammatory factors, and regulate the activity of ECE under inflammatory conditions. Enhanced ECE activity leads to increased ET-1 levels, which, as a potent vasoconstrictor involved in neuroinflammation, can aggravate neuronal damage and promote the progression of neurodegenerative diseases. Rac1 indirectly affects the clearance of Aβ by regulating the generation of ROS in astrocytes, affecting oxidative stress and the expression level of ECE. The accumulation of ROS also enhances the inflammatory response, forming a feedback loop that further weakens the neuroprotective function of astrocytes. In contrast, Cdc42 plays a key role in the migration of astrocytes and the response to injury. Its ability to regulate the formation of cellular processes promotes the migration of astrocytes to the site of injury and enhances the efficiency of Aβ clearance.

Future studies should focus on the refined analysis of the signal specificity of Rho GTPases in different pathological states and their regulatory networks, and explore strategies for the combined regulation of RhoA, Rac1, and Cdc42 to achieve optimal therapeutic effects. This will provide an important scientific basis for the development of targeted intervention methods for AD and other neurodegenerative diseases and open new directions for innovative therapies based on regulating astrocyte function.
